# Expanding the scope of medical mission volunteer groups to include a research component

**DOI:** 10.1186/1744-8603-10-7

**Published:** 2014-02-20

**Authors:** John Rovers, Michael Andreski, John Gitua, Abdoulaye Bagayoko, Jill DeVore

**Affiliations:** 1 Drake University, College of Pharmacy & Health Sciences, 2507 University Avenue, Des Moines, IA 50311, USA; 2 Drake University, College of Arts and Science, 2507 University Avenue, Des Moines, IA 50311, USA; 3 Medicine for Mali, Bacodjicoroni ACI Sud Extension, Rue 729 Porte 451, Bamako, Mali; 4 Medicine for Mali, 2683 Bryden Road, Columbus, OH 43209, USA

**Keywords:** Voluntary workers, Medical missions, Health surveys, Insecticide-treated bed nets, Malaria

## Abstract

**Background:**

Serving on volunteer groups undertaking medical mission trips is a common activity for health care professionals and students. Although volunteers hope such work will assist underserved populations, medical mission groups have been criticized for not providing sustainable health services that focus on underlying health problems. As members of a volunteer medical mission group, we performed a bed net indicator study in rural Mali. We undertook this project to demonstrate that volunteers are capable of undertaking small-scale research, the results of which offer locally relevant results useful for disease prevention programs. The results of such projects are potentially sustainable beyond the duration of a mission trip.

**Methods:**

Volunteers with Medicine for Mali interviewed 108 households in Nana Kenieba, Mali during a routine two-week medical mission trip. Interviewees were asked structured questions about family demographics, use of insecticide treated bed nets the previous evening, as well as about benefits of net use and knowledge of malaria. Survey results were analyzed using logistic regression.

**Results:**

We found that 43.7% of households had any family member sleep under a bed net the previous evening. Eighty seven percent of households owned at least one ITN and the average household owned 1.95 nets. The regression model showed that paying for a net was significantly correlated with its use, while low perceived mosquito density, obtaining the net from the public sector and more than four years of education in the male head of the household were negatively correlated with net use. These results differ from national Malian data and peer-reviewed studies of bed net use.

**Conclusions:**

We completed a bed net study that provided results that were specific to our service area. Since these results were dissimilar to peer-reviewed literature and Malian national level data on bed net use, the results will be useful to develop locally specific teaching materials on malaria prevention. This preventive focus is potentially more sustainable than clinical services for malaria treatment. Although we were not able to demonstrate that our work is sustainable, our study shows that volunteer groups are capable of undertaking research that is relevant to their service area.

## Background

Health care professionals from the developed world frequently volunteer to serve on medical mission trips to countries with large, medically underserved populations. Although some of these professionals may be paid staff members of various agencies, many are volunteers and work with small organizations. Members of volunteer groups (VGs) commonly include physicians, nurses, pharmacists, other health professionals and students [1,2]. VGs are frequently comprised of members of religious or civic groups who have an interest in health in the developing world. They raise funds, recruit volunteers, purchase medical supplies and provide medical and/or public health services. In most cases, VGs return to the same area annually to provide short-term services.

Estimates of the efforts expended by VGs suggest that the United States alone has over 500 mission organizations that send up to 6000 mission trips per year [3,4]. In 2005, there were as many as 5000 health volunteers, 1500 of whom were physicians, working in sub-Saharan Africa alone [1].

The organizations undertaking health and development work are a disparate group. Some are large, well-funded, non-governmental or academic organizations with paid staff, a global reach and a permanent presence in their service areas. Such organizations often have a research mandate as part of their mission. Other organizations are small, charity funded VGs who may only work in a single village. These smaller VGs are more typically service based and provide clinical care for only a few weeks a year.

The role, activities and limitations of these small VGs are increasingly of interest. The literature includes descriptions of the scope of their activities, personal reflections about working as a volunteer and, increasingly, critical analyses of the value of their work.

Laleman et al. found that 50-60% of the work of health volunteers in sub-Saharan Africa is clinically focused, while the remainder is often devoted to management, educating local health care workers and patients or advising on health policy [1]. Hoover et al. state that, although VGs perform a wide variety of tasks, depending on their mission, most of their efforts are clinically or administratively oriented [5]. Although some VGs may also undertake research projects relevant to their service areas, research does not seem to be central to their missions. Our literature searches have uncovered relatively few research projects performed by VGs.

Personal reflections of volunteers suggest that those who serve on medical missions view their experience quite positively and find it personally rewarding [6-9].

Positive personal perceptions notwithstanding, various authors in both the biomedical literature and the popular press raise a myriad of concerns about the value of VGs and offer recommendations for how they may improve [3,10-15]. Among the concerns raised, two major ones are the lack of sustainability of volunteer work and a focus on treating acute, often self-limited problems, rather than on preventing the underlying causes of more serious diseases [10]. Short term medical volunteers are also criticized for creating dependency upon their services, thus absolving foreign governments from creating adequate health care systems [13]. Other authors have noted that what they call medical “voluntourists” are often not well prepared, either clinically or culturally, to work in developing world settings [3,15]. VGs may not consider the ethical ramifications of their work and realize they are affecting the lives of vulnerable people [11,12]. Other authors express concerns about the quality of care VGs may provide [14].

Given that they commonly work in sub-Saharan Africa and other areas where malaria is endemic, it is not surprising that VGs often care for malaria patients. Malaria remains a significant problem, despite VGs’ provision of demonstrably effective preventive measures such as insecticide-treated bed nets (ITNs) and efficacious pharmacotherapy, Although between 2000 and 2010, malaria mortality decreased by 26%, and an estimated 1.1 million deaths were prevented, the 2012 World Malaria Report indicates that in 2010, there were 219 million cases of malaria and 660,000 deaths [16]. Given the enormity of this disease burden, it is not surprising that a plethora of organizations include malaria-related work as part of their mission.

### Objective

In this paper, we hope to reconcile the three issues raised above, namely the lack of research focus by VGs, criticism that VGs’ services lack a sustainable focus and treat only acute illnesses, and the burden caused by malaria in the developing world. Our hypothesis is that VGs can expand the scope of their missions to include small-scale research activities that focus specifically on important health problems in the communities they serve. This research, in turn, is potentially useful for VGs to create and provide services that are sustainable beyond the duration of a short-term medical mission and are useful for preventing disease.

Here, we report our experience of a small VG undertaking a survey of ITN use in a Malian village. Our objective is to demonstrate that VGs can perform locally relevant research and that such research has implications for health services lasting beyond acute illnesses and a short-term mission trip.

It should be noted that it is not our intention to publish the complete results of our ITN survey, as the results are applicable only to the service area of our VG and the literature is already replete with large, well-performed ITN studies.

## Methods

### Study setting

Since 2000, a small VG, Medicine for Mali, has provided a variety of health and development services in the Cercle of Kati, located in the Koulikoro Region of southern Mali [17]. Medicine for Mali relies primarily on volunteer health workers (both Malian and American) and donations to undertake its work.

Historically, Medicine for Mali’s focus has been on service provision, not research. Medicine for Mali provides a broad variety of clinical and public health services. In addition to an annual medical clinic, other services include programs in drilling clean water wells, public health education, microfinance (especially for village women), teacher training, and scholarships for village children to attend high school in the capital city, Bamako.

A Medicine for Mali team usually consists of 12–15 individuals. About a third are health care providers in private practice. Another third are medical or physician assistant students and faculty from American medical or pharmacy schools. The remainder of the team are aid workers, primarily for the microfinance, clean water, public health and education programs who may be on site for up to six weeks. A typical Medicine for Mali medical mission occurs for two weeks each February, concurrent with the lengthier mission of the public health and microfinance workers.

In addition to American volunteers, Medicine for Mali employs a Malian physician to manage its programs year round, when no volunteers are in the village. Three villagers volunteer to serve as community health workers and are trained by Medicine for Mali to provide education on a variety of public health topics. During each mission trip, local Malians are hired to be interpreters.

The survey was carried out in the village of Nana Kenieba, Mali. Medicine for Mali workers estimate the population to be approximately 600 persons since there is no reliable census data. The village is in the Cercle of Kati, located in the Koulikouro Region (approximately 12°20′N by 8°20′W) southwest of the capital city, Bamako. Villagers are primarily subsistence farmers and poverty is endemic to the region. There is no electricity in the village and cell phone service is occasionally accessible by climbing a hill on the edge of the village where there is a line of sight to the nearest cell phone tower in the neighboring market town of Siby.

Nana Kenieba is located in an area of high malaria transmission [16]. Although malaria is seen year round, the climatic conditions most suitable for malaria transmission are between June and October [18,19]. The survey was conducted during the annual Medicine for Mali mission trip in February 2012, at a time of year when malaria transmission is lower than during the rainy season. Shortly after our trip, Mali suffered both a military coup and terrorist incursions. The unstable political situation precluded a trip in 2013, however there are plans for a small team to travel to Nana Kenieba in 2014.

### Survey

The survey instrument was designed using the recommendations of the Malaria Indicator Surveys developed by Roll Back Malaria, as well as the malaria related questions on the Multiple Indicator Cluster Surveys used by UNICEF [20,21]. Structured questions inquired into family demographics and education, ITN ownership, indoor residual spraying, ITN use the previous night, reasons for not sleeping under an ITN, benefits of sleeping under an ITN, intermittent protective therapy in pregnant women, the respondent’s knowledge of malaria and a visual inspection of any nets seen in the household.

### Sampling procedures

We assumed that Nana Kenieba had a population of 600 and that the average household had five to six members, which suggests a village of 100–120 households. Accordingly, we planned on surveying all village households during the trip.

### Local participation

As the protocol for this study was being prepared, the American investigators communicated by email with the local Malian physician about project goals and methods. We requested that he discuss the project with village elders and familiarize them with the project. Upon arrival in Nana Kenieba, two Medicine for Mali team members and the Malian physician met with the village elders to discuss the project and seek their approval to undertake the survey. Village elders commented that Medicine for Mali had a long history of providing service to the village and gave their approval for the survey.

In addition to a paid translator to assist with interviews, a local villager volunteered to act as a guide around the village. At each household, he introduced the Medicine for Mali team members, explained the project and confirmed that the village elders had given their approval.

### Ethical approval

The study was approved by the Drake University Institutional Review Board. Since the literacy of the villagers and the need to translate a consent form into the local language (Bambara) were problematic, the translator explained the survey, including risks and benefits to villagers who gave their verbal assent to participate in the study.

### Statistical methods

The dependent outcome variable, “Did anyone in this house sleep under a bet net last night?”, was a measure composed of the five questions on the survey, where each question asked if certain household members (e.g. children under 5 years) had slept under a bed net the previous night. The five questions encompassed all possible household members. For purposes of analysis this was converted to a binary dependent variable of “Yes” or “No”.

Two binary independent variables were constructed from two questions on the survey that were measured by a Likert-type scale. These variables were “greater than 4 years of school for the male head of household” and “more than 2 nets owned by the household”. The other independent variables tested were: belief in not getting bitten by mosquitoes as the major benefit of sleeping under an ITN; belief in not getting malaria as the major benefit of sleeping under an ITN; net age of less than one year; ITN obtained from the public sector; having to pay for the ITN; absence of mosquitos as rationale for not sleeping under an ITN; house being treated by indoor residual spraying with insecticide; and presence of visible holes in observed ITNs.

Multiple logistic regression models were built to determine the effect of each of the independent variables on the outcome measure, while controlling for confounding effects of non-significant variables. A forward step-wise technique was used in the determination of models. All statistical analysis was performed using IMB Statistics SPSS 20.

## Results

A total of 108 households were surveyed during the mission trip. Information on ITN usage was available for 103 households. The average number of individuals in the households was 5.46 (±2.67). The average number of years of schooling for the male head of household was 3.86 (±3.86) years, and 3.77 (±3.45) years for the female head of household. Table 1 contains further information about the composition of the households studied. Of the 103 households in which information was available, 45 (43.7%) had at least one person who had slept under an ITN the previous night. Eighty seven percent of households owned at least one ITN and the average household owned 1.95 nets.

**Table 1:** Characteristics of studied households

	**Mean (±SD)**	**Proportion of households containing**
Males > 15 y.o.	1.09 (±0.45)	96.1%
Females > 15 y.o.	1.41 (±0.82)	97.1%
Pregnant females > 15 y.o.	0.11 (±0.31)	10.9%
Children 5–15 y.o.	1.94 (±1.74)	71.8%
Children < 5 y.o.	1.02 (±1.05)	38.8%

SD: Standard Deviation.

A model to determine factors that may predict the likelihood of someone in the household having slept under an ITN the previous night was determined using a logistic regression technique. A model that included five independent variables plus a constant correctly predicted use of an ITN the previous night in 85% of cases.

Households that had paid for their nets were significantly more likely to have slept under a net the previous night. Households that reported that they did not see mosquitos, had obtained their net from a public source, and had a male head of household with over four years of school were significantly less likely to have done so. Table 2 contains further information about the components of the model.

**Table 2:** Multivariable analysis of likelihood of someone in the household sleeping under a bed net the previous night

	**OR**	**(95% CI)**	**p-value**
Greater than 4 years of school for the male head of household	0.242	(0.56-1.052)	.058
Net age of less than 1 year	2.271	(0.327-15.776)	.407
Net obtained from the public sector	0.133	(0.22-0.795)	.027
Having to pay for the net	6.106	(0.796-46.843)	.082
Absence of mosquitos	0.039	(0.008-0.197)	.000
Constant	22.352		.001

OR: Odds Ratio CI: Confidence Interval.

Of the 695 patients treated over a two-week trip, five were diagnosed as having malaria using the Malaria P.f. rapid diagnostic test.

## Discussion

Our results demonstrate that VGs are capable of performing small-scale research studies targeted to the communities they serve. Other VGs who have undertaken research on their activities limited their studies to retrospective descriptions of the demographics of their patient population and the diseases treated.

Niska and Sloand found that most patients seen during a mission trip to Haiti were females over age 15 [7]. Children most commonly suffered from intestinal parasites, while adults’ most common complaints were gastritis and musculoskeletal pain. Martiniuk et al. examined the health records of 2500 Honduran patients served by a VG. Infectious and parasitic diseases were seen to be the most common complaints [22].

Our study appears to be unique for two reasons: (1) data was collected prospectively, during a routine medical mission trip; (2) our results are potentially useful to design educational programs for malaria prevention by promoting ITN use. Although the results of Niska and Sloand [7] and Martiniuk et al. [22] would be useful to determine what kinds of patients would be seen and medications needed on future trips to Haiti or Honduras, our results would be more helpful in planning ITN education programs for community health workers and villagers. As such, our results appear to be more likely to be potentially sustainable and focused on prevention, rather than treatment.

We surmise that an existing, positive relationship with the villagers of Nana Kenieba was beneficial to performing our study. As we introduced our project to the village elders, they expressed considerable support. They gave the long-standing relationship between Nana Kenieba and Medicine for Mali as a major reason for their cooperation. Once we began data collection, one of the villagers volunteered, unasked and unpaid, to guide us around the village and introduce us to interview subjects. When we asked villagers to participate, their participation was universal and interpretable data was obtained for 103 out of 108 potential households surveyed. Other authors confirm that a good working relationship between aid workers and villagers is necessary to gain local support for a project like this [23-26].

Although the literature is replete with studies of ITN use, they often employ research methods beyond the capabilities of VGs. Some are qualitative studies that may be time consuming or require special training in qualitative methods [27]. Others utilized complex cluster sampling techniques or included thousands of subjects spread across multiple villages or regions [28-30]. Such methods can be expensive and beyond the means of a VG.

Even more problematic, some studies suggest that ITN use is locally variable. The reasons why individuals may or may not sleep under an ITN can depend on where they live [24,31,32]. Since VGs often work in a very limited geographic area, studies performed in a local area may produce results that are more useful in designing education programs that actually reflect ITN use in the VGs’ service area.

For Mali, the 2012 World Health Report Country Data Sheet indicates that in 2011, approximately 65% of Malians slept under an ITN [16]. The report also states that 90% of Malians live in an area of high transmission, defined as ≥ 1 case per 1000 population. This data is different from our results, which show that 43.7% of households in our sample had any family member sleep under an ITN the previous night. And since we treated five confirmed cases of malaria out of 695 patients seen, it appears that the risk of malaria in Nana Kenieba is quite high, despite it being the dry season. The World Health Report Country Data Sheet also describes ITN ownership models suggesting that 60% of Malian households own an ITN. This is lower than the 87% ITN ownership seen in our study. Although regional or national level data may be all that is available, there appears to be a potential for VGs to create their own datasets that are more valuable than larger ones. It appears that in Nana Kenieba, malaria risk is high, net ownership is higher than national data but net use is lower than national data. This information can be used to create malaria education programs for villagers that reflect the specific conditions seen locally.

Related to the local explanations for ITN use is that the literature shows the stated reasons for not sleeping under an ITN are inconsistent. Reasons may vary according to study methodology, geographical factors or a person’s knowledge about malaria. Studies show that reasons may include perceptions of low mosquito density, changes in sleeping arrangements, difficulties in physically hanging ITNs, feeling hot or uncomfortable and a variety of other reasons [27-30,33-43]. Our literature searches did not reveal any peer-reviewed literature on factors that influence ITN use in Mali, thus making our locally derived data especially relevant. Given the variability in ITN use by locale, and the diverse explanations for ITN use in the literature, it does seem plausible that VGs that can gather local results and identify factors underlying ITN use may be in a better position to develop ITN education programs than VGs that depend on published or government data.

### Limitations

Our contention that VGs are capable of small-scale research projects may be limited by the particular membership on a medical mission team. Even a very local study using simple data collection and analysis methods requires at least one team member with basic skills in study design, data collection and statistical analysis if the study results are to have any meaning. Although not every VG will include such team members, the literature suggests that VG teams frequently do include clinical faculty from medical, pharmacy or nursing schools who either have, or have access to, the research skills necessary to undertake a basic study [2,11,13]. In the case of our study, even though the research members of the team were based in universities that emphasize teaching over research, we had little difficulty designing a study and data analysis plan.

We would also note that our survey was performed during the dry season when transmissibility of malaria is lower and fewer mosquitos may be seen. This may have influenced subjects’ responses when answering survey questions.

Our hypothesis that VGs’ research can be sustainable beyond the duration of a mission trip remains unproven. The political situation in Mali precluded a return visit in 2013 to discuss the results of the ITN survey with village leadership and health workers. However, planning for a small team to return in 2014 is well underway and the results of our survey will be used to educate villagers on bed net use and malaria prevention. Since our results suggest paying for an ITN was positively correlated with its use, we may also have to review our policies for distributing ITNs. It appears that the size and resources of a small VG like ours pose limits on how rapidly a research project can identify a problem, propose and implement a solution and assess the effectiveness of the intervention.

## Conclusions

We conclude that an appropriately constituted VG is capable of performing small-scale research, the results of which may be more applicable to the local situation in the communities they serve than regional or national data collected by larger organizations. The VG should have an existing relationship with the community in which it intends to do the study and the trust of the villagers. A well-chosen research objective and methodology, which are consistent with the capabilities of the VG, are necessary for success. Having at least one team member with research skills, or access to researchers in their home country is necessary.

## Abbreviations

VG: Volunteer group; ITN: Insecticide treated bed net.

## Competing interests

The authors declare that they have no competing interests.

## Authors’ contributions

JR originally conceptualized the study. JR and MA designed the methods with contributions from all other authors. JG performed the survey interviews and collected the data with assistance from JR. AB and JD provided logistic support and coordination between the USA and Mali. MA performed the statistical analysis with assistance from JR. JR wrote the manuscript with the exception of the statistical methods and results sections, which were written by MA. All authors have reviewed and approved the final manuscript.
